# Supplementary material for: The interactome of intact mitochondria by cross-linking mass spectrometry provides evidence for coexisting respiratory supercomplexes
Source: Mol Cell Proteomics. 2017 Dec 8;17(2):216–32. doi: 10.1074/mcp.RA117.000470 (PMC5795388; doi:10.1074/mcp.RA117.000470)
Supplement: Supplemental Data [file supp_RA117.000470_133922_0_supp_23978_fzffwf.zip › spectra_annotation/mito_spectra_annotation/334-1-4-1-10-1.pdf]

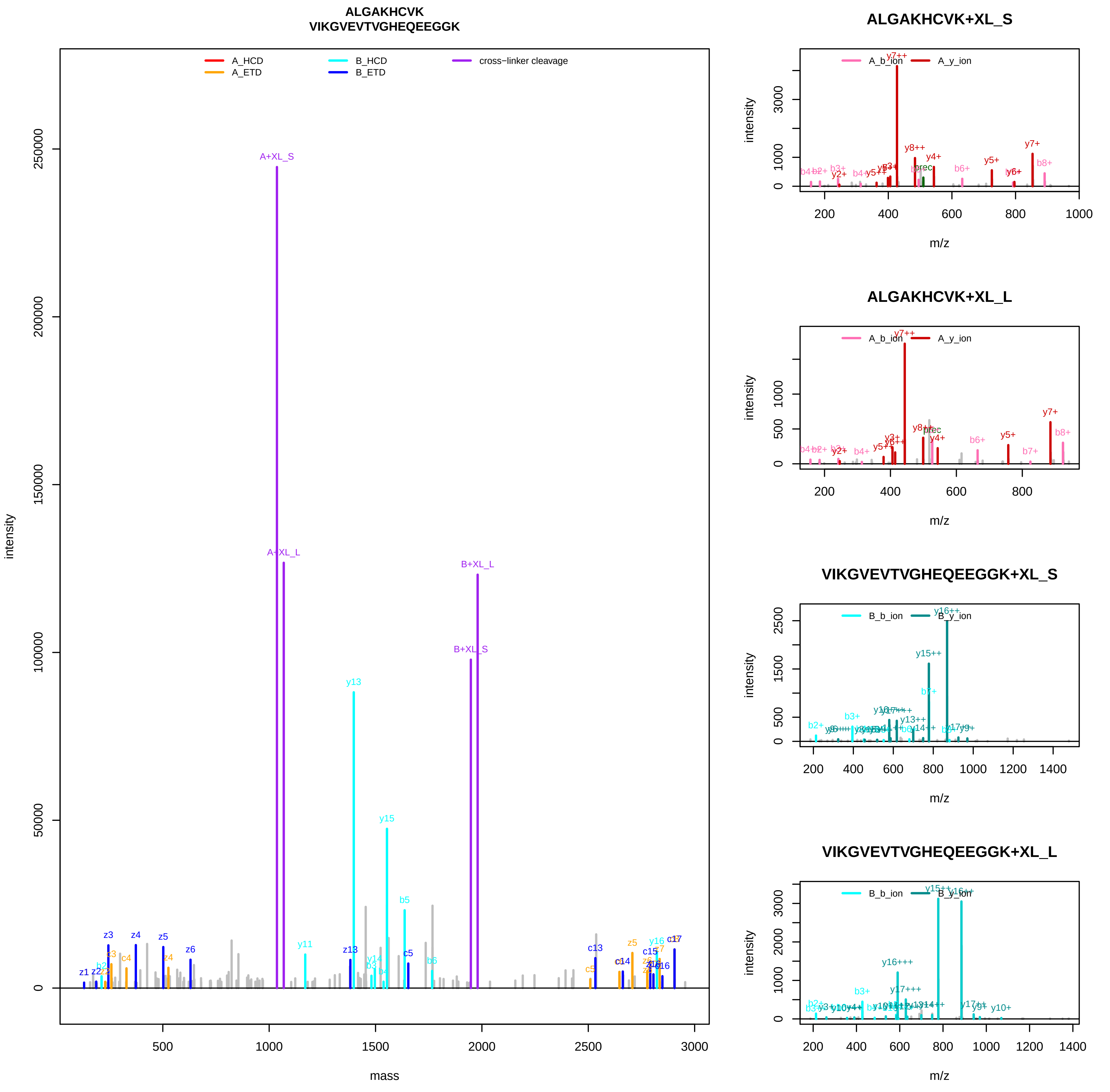
ALGAKHCVK
VIKGVEVTVGHEQEEGGK
A_HCD
A_ETD
B_HCD
B_ETD
cross−linker cleavage
A+XL_S
A+XL_L
B+XL_L
B+XL_S
y13
y15
y11
b5
z13
c5
c13
c14
c15
c17
z5
y16
z1
z2
z3
z4
z5
z6
c3
c4
intensity
mass
0
50000
100000
150000
200000
250000
500
1000
1500
2000
2500
3000
ALGAKHCVK+XL_S
A_b_ion
A_y_ion
y7++
y8++
y7+
y5+
b8+
b6+
prec
m/z
ALGAKHCVK+XL_L
b7+
VIKGVEVTVGHEQEEGGK+XL_S
B_b_ion
B_y_ion
y16++
y15++
b7+
b3+
b2+
y13++
VIKGVEVTVGHEQEEGGK+XL_L
y15++
y16+++
y17+++
b3+
b2+
y10+
